# Disparities in Access to Autologous Breast Reconstruction

**DOI:** 10.3390/medicina56060281

**Published:** 2020-06-08

**Authors:** David J. Restrepo, Maria T. Huayllani, Daniel Boczar, Andrea Sisti, Minh-Doan T. Nguyen, Jordan J. Cochuyt, Aaron C. Spaulding, Brian D. Rinker, Galen Perdikis, Antonio J. Forte

**Affiliations:** 1 Division of Plastic Surgery, Mayo Clinic, Jacksonville, FL 32224, USA; rpo20@hotmail.com (D.J.R.); maria.t.huayllanip@gmail.com (M.T.H.); danielboczar92@gmail.com (D.B.); rinker.brian@mayo.edu (B.D.R.); 2 Department of Plastic Surgery, Cleveland Clinic, OH 44195, USA; asisti6@gmail.com; 3 Division of Plastic Surgery, Mayo Clinic, Rochester, MN 55905, USA; Nguyen.Minh-Doan@mayo.edu; 4 Department of Health Science Research, Mayo Clinic, Jacksonville, FL 32224, USA; cochuyt.jordan@mayo.edu (J.J.C.); Spaulding.Aaron@mayo.edu (A.C.S.); 5 Department of Plastic Surgery, Vanderbilt University Medical Center, Nashville, TN 37232, USA; galen.perdikis@vanderbilt.edu

**Keywords:** breast cancer, breast reconstruction, autologous reconstruction, disparities, public health, Florida

## Abstract

*Background and objectives:* This study aimed to determine if age, race, region, insurance, and comorbidities affect the type of breast reconstruction that patients receive. *Materials and methods:* This analysis used the Florida Inpatient Discharge Dataset from 1 January 2013 to 30 September 2017, which contains deidentified patient-level administrative data from all acute care hospitals in the state of Florida. We included female patients, diagnosed with breast cancer, who underwent mastectomy and a subsequent breast reconstruction. We performed an χ^2^ test and logistic regression in this analysis. *Results:* On the multivariable analysis, we found that age, race, patient region, insurance payer, and Elixhauser score were all variables that significantly affected the type of reconstruction that patients received. Our results show that African American (odds ratio (OR): 0.68, 95%CI: 0.58–0.78, *p* < 0.001) and Hispanic or Latino (OR: 0.82, 95%CI: 0.72–0.93, *p* = 0.003) patients have significantly lower odds of receiving implant reconstruction when compared to white patients. Patients with Medicare (OR: 1.57, 95%CI: 1.33–1.86, *p* < 0.001) had significantly higher odds and patients with Medicaid (OR: 0.61, 95%CI: 0.51–0.74, *p* < 0.001) had significantly lower odds of getting autologous reconstruction when compared to patients with commercial insurance. *Conclusions:* Our study demonstrated that, in the state of Florida over the past years, variables, such as race, region, insurance, and comorbidities, play an important role in choosing the reconstruction modality. More efforts are needed to eradicate disparities and give all patients, despite their race, insurance payer, or region, equal access to health care.

## 1. Introduction

The United States has 3.5 million breast cancer survivors [1]. With an estimated 279,100 new breast cancer diagnoses for 2020 and a steady decrease in mortality, the number of survivors is expected to increase [2,3].

Although lifesaving, mastectomy is a procedure that can cause significant psychological stress in patients who require it [4]. To improve this burden, breast reconstruction has become an important source of hope for these women. Women who receive breast reconstruction have shown improvement in health-related quality of life [4], while sexuality has been shown to be worse in patients who do not receive postmastectomy breast reconstruction [5].

As breast reconstruction rates increase [6], and with laws that make the procedure more widely available to the US population [7], new procedures have been developed and have proven to be as good, or better, than previous procedures. Breast reconstruction techniques currently available can be divided into autologous tissue-based or implant-based techniques [8]. Implant-based reconstruction is the most common type of reconstruction; however, women who receive autologous reconstruction have shown a higher rate of satisfaction [9].

The type of reconstruction used for a patient who received a mastectomy should be based on multiple factors; patient preference, age, weight, comorbidities, shape and size of the breast, mastectomy scar, surgeon experience, and cost should all be taken into account when selecting a reconstructive technique. However, factors, such as race, region, and insurance, should not be factors that affect the type of reconstruction. Equality is one of the main goals of the healthcare system, and disparities should not be present in any aspect of medicine. It has been reported that African American race is the most clinically significant predictor of autologous breast reconstruction and there is little data regarding the Hispanic population. With this study, we aimed to analyze the Florida Inpatient Discharge Dataset (FIDD) to see if factors, such as age, race, region, insurance, and comorbidities, have an effect on the type of breast reconstruction received by postmastectomy patients in Florida.

## 2. Materials and Methods

### 2.1. Data Source

This analysis used the FIDD, which contains deidentified patient-level administrative data from all acute care hospitals in the state of Florida.

### 2.2. Population and Variables

**Figure 1:**
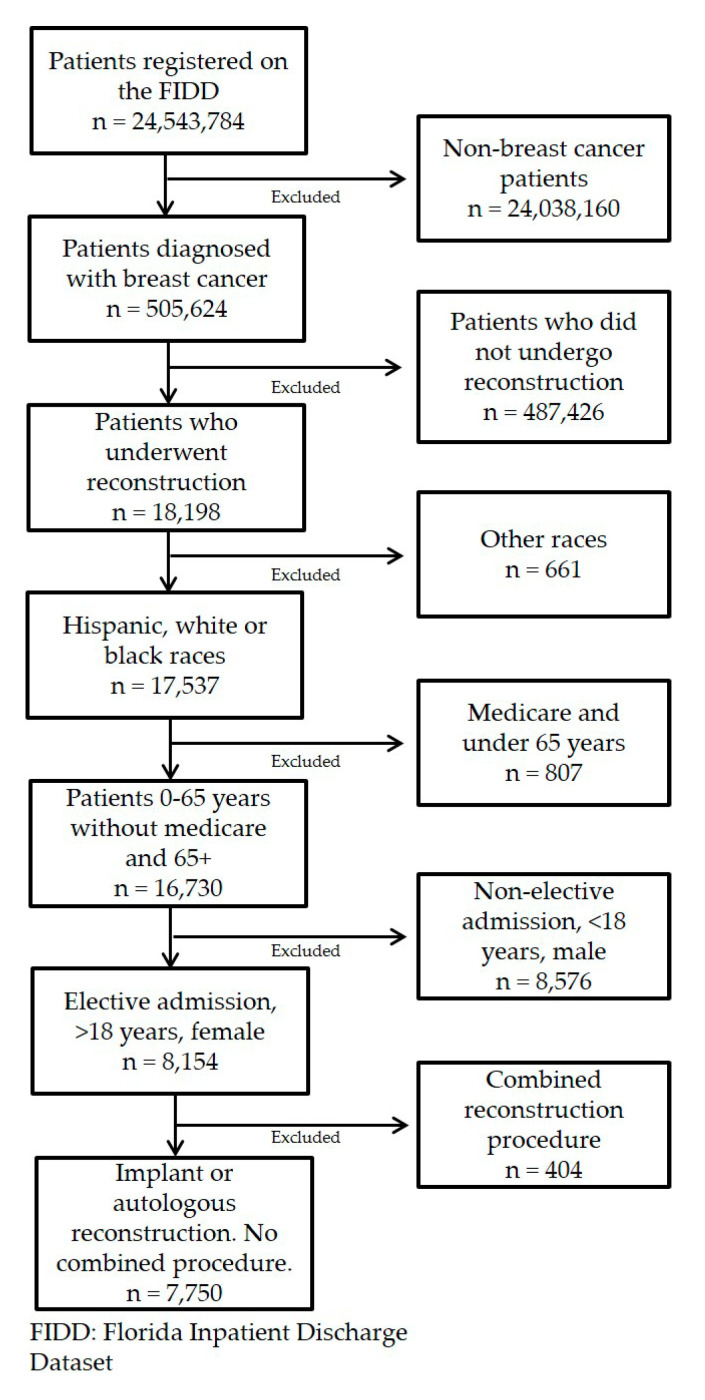
Inclusion and exclusion criteria.

We included female patients who were 18 years or older, diagnosed with breast cancer, who underwent an elective reconstructive surgery involving either an implant or an autologous procedure from 1 January 2013 to 30 September 2017. We excluded male patients, and subjects who were enrolled on Medicaid but were less than 65 years old. The inclusion and exclusion of our population is further depicted in Figure 1.

### 2.3. Dependent Variables

The dependent variable for this analysis was whether the patient had an implant procedure or an autologous procedure (i.e., free, pedicled). The surgical procedures were defined by the International Classification of Diseases-9 (ICD) and ICD-10 codes.

### 2.4. Independent Variables

Patient characteristics, including age, race/ethnicity, region, insurance payer type, and comorbidities, were included as covariates. Race and ethnicity were categorized as white, black or African American, and Hispanic or Latino. Insurance payer type was categorized as Medicare (including Medicare Managed Care Patient), Medicaid (including Medicaid Managed Care Patient), commercial, or other (including self-pay or non-payment). Patients’ regional locations were based on the seven regions of the Florida Department of Transportation, and indications of rurality were defined by the Florida Department of Health. We collapsed patient regions into North, South, and Central to allow for an acceptable amount of statistical power for the multivariable models. The Elixhauser score was used to indicate whether patients had comorbidities.

### 2.5. Analysis

Data were described as frequency and percentage or median and range. Pearson χ^2^ and Kruskal–Wallis tests were used to compare categorical and continuous variables. The first statistical model focused on disparities among patients who received an implant or a flap procedure. We used multivariable logistic regression and summarized the data using odds ratios (ORs). Furthermore, 95% Confidence Intervals (CIs) were used to show the strength of the association between each of the different comparisons. All tests of significance were two-sided, and *p* values were reported. The level of statistical significance was set at α less than 0.05. Analyses were preformed using SAS, version 9.4 (SAS Institute Inc. Cary, NC).

## 3. Results

A total of 7750 patients underwent postmastectomy breast reconstruction during the study period and met our inclusion criteria for the study. Table 1 outlines their demographic characteristics and Elixhauser Comorbidity Index score by type of reconstruction. Significant differences were found in all the groups. In our cohort, 4944 (68.7%) were white, 882 (12.3%) African American, and 1368 (19.0%) Hispanic or Latino. While most patients (7080 (95.1%)) had insurance, (361 (4.9%)) did not. There were a higher number of patients with no comorbidities (4185 (56.2%)) than patients with at least one comorbid condition (3256 (43.8%)), as calculated with the Elixhauser score.

Using multivariable analysis, we found that age, race, patient region, insurance payer, and Elixhauser score were all variables that significantly affected the type of reconstruction that patients received (Table 2). The results show that both African American (OR: 0.68, 95%CI: 0.58–0.78, *p* < 0.001) and Hispanic or Latino (OR: 0.82, 95%CI: 0.72–0.93, *p* = 0.003) races have significantly lower odds of receiving implant reconstruction when compared to white patients. Interestingly, insurance showed mixed results; patients with Medicare (OR: 1.57, 95%CI: 1.33–1.86, *p* < 0.001) had significantly higher odds while patients with Medicaid (OR: 0.61, 95%CI: 0.51–0.74, *p* < 0.001) had significantly lower odds of getting implant reconstruction when compared to patients with commercial insurance. Patients with comorbidities, defined as an Elixhauser score greater than 0, were also found to have lower odds of implant reconstruction when compared to patients without comorbidities (OR: 0.80, 95%CI: 0.72–0.88, *p* < 0.001).

Our multivariable analysis comparing the two types of autologous reconstruction (flap and pedicled flap) versus implant-based reconstruction alone also showed significant associations between the studied variables (Table 3). In terms of race, African Americans showed lower odds of getting implant reconstruction versus any type of flap when compared to white patients (implant versus free flap (OR: 0.61, 95%CI: 0.51, 0.74, *p* < 0.001) and versus pedicled flap (OR: 0.61, 95%CI: 0.44, 0.88, *p* = 0.007). On the contrary, there was no significant difference for Hispanic or Latino patients in implant versus pedicled flap reconstruction (OR: 0.96, 95%CI: 0.70, 1.31, *p* = 0.79) when compared to white patients.

## 4. Discussion

Breast cancer incidence is increasing [3]. Throughout their lifetime, breast cancer will affect one in every eight women in the United States [10]. Although breast conservation therapy remains an option, many of these women instead undergo mastectomy with or without breast reconstruction [11,12,13,14,15]. Efforts have been made nationally to make breast reconstruction more available to patients who undergo mastectomy. Evidence has shown an increase in the rate of breast reconstruction, which is a step forward in the physical and psychological treatment of breast cancer [16]. However, there are few studies of the disparities in access to autologous breast reconstruction in the United States [11,17,18,19], and none in Florida, where there is a larger Hispanic population than in most of states. The importance of this study is that it addresses this gap in the literature and will aid public health agencies to understand the factors that influence access to autologous breast reconstruction.

Despite not having a consensus on whether implant- or autologous-based reconstruction is better for patients, autologous-based reconstruction is currently recognized as the best option by providing the patient with a more natural look and feel [8]. When it comes to selecting the type of reconstruction a patient should receive, race, region, and insurance should not be determining factors.

Our results show that, even when corrected for confounders, some of these variables were significant when deciding what type of reconstruction patients received. Minorities, such as African American or Hispanic (Latino), had lower odds of receiving implant breast reconstruction, which implies that they received more autologous reconstruction. These results were expected, since despite lower rates of postmastectomy breast reconstruction in black patients when compared to white patients [17], it has already been shown that black patients have a higher rate of autologous breast reconstruction [11,17,18].

Sergesketter et al. [19] reported that black race (non-Hispanic) and Hispanic ethnicity had a lower likelihood of receiving breast reconstruction when compared to white patients. However, they also found that these two groups of patients were more likely to receive autologous than implant-based reconstruction.

Moreover, three studies found that black patients had a higher rate of autologous breast reconstruction when compared to their white counterparts [11,17,18]. One study found no significant difference between the groups [20].

Our results are in concordance with these studies and further contribute to the available literature on this subject. However, none of these studies identified the causality for these results. Unfortunately, disparities affecting breast cancer patients are not limited to autologous reconstruction. Our group has shown that the rate of breast reconstruction, refusal of surgical treatment, and the survival of male patients with breast cancer has also been shown to be affected by ethnic and demographic characteristics [21,22,23,24].

It was reported in two studies that postmastectomy breast reconstruction decreases the probability of depression and improves emotional, social, and physical functionality. Women who do not receive postmastectomy breast reconstruction have worse functionality on the mentioned aspects [5,25]. Although implant-based reconstruction is more common, a recent study published by Fracon and colleagues [9] showed that women who received autologous breast reconstruction showed a higher degree of satisfaction using the BREAST-Q module (*p* = 0.00596), psychosocial well-being module (*p* = 0.04), and sexual well-being module (*p* = 0.00068).

Despite having a longer operating time and more incisions, autologous breast reconstruction, such as the deep inferior epigastric (DIEP) flap, has been found to have fewer serious adverse effects leading to reconstruction failure or unpleasant aesthetic results when compared to tissue expander implant-based reconstruction [26].

Interestingly, Fischer and colleagues [27] conducted a study in which adverse effects were compared in patients receiving free flap reconstruction versus tissue expander and implant reconstruction in a high-volume institution. On an average two-year follow-up, a 98.8% success rate was reported with free flap reconstruction, while a 94.4% success rate was reported with tissue expander reconstruction. Interestingly, the free flap was also associated with a lower rate of unplanned reoperation (5.8% vs. 16.8%; *p* = 0.002) [27]. These results are in line with Spear and colleagues’ results [28]. In contrast, Mioton and colleagues [29], using the National Surgical Quality Improvement Project Dataset, reported higher rates of failure in autologous reconstruction (3.13% vs. 0.85%; *p* < 0.001); however, it is important to note that the follow-up period included only 30 days, showing that during the first 30 days, autologous reconstruction can present a higher rate of failure. Autologous reconstruction is also known to be a more complicated procedure, requiring a longer operating time. Due to the higher complexity of autologous breast reconstruction, its costs are higher. However, Fischer and colleagues [27] reported that even though autologous reconstruction has a higher upfront operation cost, it is more cost efficient over time. Other studies have supported this result, especially over time [30,31].

Surgeon reimbursement can be a matter of discussion, too, as it is sometimes believed that autologous reconstruction is not cost effective for practitioners. Sando and colleagues [32] reported that contrary to the perceptions, the complex reconstructive procedures that patients undergo for autologous reconstruction consistently generated more revenue and an hourly reimbursement that showed no statistical difference ($1053 vs. $947; *p* = 0.72). Other studies have demonstrated that autologous reconstruction is also more cost-efficient and profitable for hospitals [29,33].

Despite all this information, implant reconstruction is more common than autologous reconstruction. National trends show an increase in implant-based breast reconstruction and a decrease in autologous reconstruction [34]. However, plastic surgeons who practice at academic university programs do not follow the same trends [34]. Implant-based reconstruction rates, which have typically trended up, now show a plateau in academic hospitals, while the rate of autologous reconstruction, more specifically the DIEP flap, has increased [34].

Considering the previously mentioned information in favor of autologous breast reconstruction, it is interesting to see that in Florida, black and Hispanic patients receive a lower proportion of implant reconstructions, suggesting a higher rate of autologous reconstruction. It was also shown that patients with Medicaid who receive reconstruction have lower odds of getting implant reconstruction, implying a higher proportion of autologous reconstruction when compared to commercial insurance. On the contrary, patients with Medicare had lower odds of receiving autologous breast reconstruction, showing that there are disparities, even among the two government insurance types.

This study has several limitations. Our analysis was done using the FIDD because it captures 100% of the patients with breast cancer treated in Florida health institutions. Due to the nature of databases, the fidelity of the information can be affected by incorrect or incomplete reports. Furthermore, the FIDD registers every event separately, meaning that if the same patient has two outpatient visits or two different reconstruction procedures, her cases would be registered as two separate cases. Additionally, a large proportion of the patients in the database were excluded due to our inclusion and exclusion criteria; however, the number of studied patients was substantial and allowed for multivariable analyses.

## 5. Conclusions

Our study demonstrated that, in the state of Florida, variables, such as race, region, insurance, and comorbidities, seem to play an important role in selecting the reconstruction modality. More efforts are needed to eradicate disparities and give all patients, despite their race, insurance, or region, equal access to health care.

## Figures and Tables

**Table 1:** Surgical population’s descriptive statistics for breast cancer patients.

Variable	Flap (n = 2809)	Implant (n = 4632)	Total (n = 7441)	*p* Value
Age	54.0 (20.0–93.0)	53.0 (21.0–87.0)	53.0 (20.0–93.0)	<0.0001 ^1^
Year				<0.0001 ^2^
2013	752 (26.8%)	1319 (28.5%)	2071 (27.8%)	
2014	765 (27.2%)	1147 (24.8%)	1912 (25.7%)	
2015	685 (24.4%)	1004 (21.7%)	1689 (22.7%)	
2016	382 (13.6%)	821 (17.7%)	1203 (16.2%)	
2017	225 (8.0%)	341 (7.4%)	566 (7.6%)	
Race				<0.0001 ^2^
White	1798 (66.2%)	3146 (70.2%)	4944 (68.7%)	
Black or African American	400 (14.7%)	482 (10.8%)	882 (12.3%)	
Hispanic or Latino	517 (19.0%)	851 (19.0%)	1368 (19.0%)	
Patient Region				
South Florida	1257 (46.1%)	2465 (54.3%)	3722 (51.2%)	<0.0001 ^2^
North Florida	605 (22.2%)	781 (17.2%)	1386 (19.1%)	
Central Florida	867 (31.8%)	1294 (28.5%)	2161 (29.7%)	
Patient Insurance Payer				<0.0001 ^2^
Medicare	479 (17.1%)	839 (18.1%)	1318 (17.7%)	
Medicaid	238 (8.5%)	267 (5.8%)	505 (6.8%)	
Commercial	1916 (68.2%)	3341 (72.1%)	5257 (70.6%)	
Other	176 (6.3%)	185 (4.0%)	361 (4.9%)	
Elixhauser Score				<0.0001 ^2^
No	1445 (51.4%)	2740 (59.2%)	4185 (56.2%)	
Yes	1364 (48.6%)	1892 (40.8%)	3256 (43.8%)	

Statistical tests of difference: ^1^ Kruskal–Wallis, ^2^ χ2. Statistics reported: Continuous variables were summarized with the median (range).

**Table 2:** Odds ratio of receiving implant breast reconstruction.

	Implant vs. Flap (Ref)	
Variable	OR (95%CI)	*p* Value
Age (10-year increase)	0.79 (0.75, 0.84)	<0.0001
Race	Overall Test of Difference: *p* < 0.0001	
White	1.00 (Ref)	N/A
Black or African American	0.67 (0.57, 0.78)	<0.0001
Hispanic or Latino	0.81 (0.71, 0.92)	
Patient Region	Overall Test of Difference: *p* < 0.0001	
North Florida	1.00 (Ref)	N/A
South Florida	1.61 (1.40, 1.83)	<0.0001
Central Florida	1.17 (1.02, 1.35)	
Patient Insurance Payer	Overall Test of Difference: *p* < 0.0001	
Commercial	1.00 (Ref)	N/A
Medicare	1.59 (1.34, 1.89)	<0.0001
Medicaid	0.62 (0.51, 0.75)	<0.0001
Other	0.64 (0.51, 0.80)	
Elixhauser Score		
No	1.00 (Ref)	N/A
Yes	0.75 (0.68, 0.82)	<0.0001

Abbreviations: N/A, not applicable; OR, odds ratio; Ref, reference.

**Table 3:** Logistic regression comparing different types of autologous vs. implant reconstruction.

	Implant vs. Free Flap (Ref)		Implant vs. Pedicled Flap (Ref)	
Variable	OR (95%CI)	*p* Value	OR (95%CI)	*p* Value
Age	0.81 (0.75, 0.88)	<0.0001	0.63 (0.54, 0.72)	<0.0001
Race	Overall Test of Difference: *p* < 0.0001		Overall Test of Difference: *p* = 0.009	
White	1.00 (Ref)	N/A	1.00 (Ref)	N/A
Black or African American	0.61 (0.51, 0.74)	<0.0001	0.61 (0.44, 0.84)	0.003
Hispanic or Latino	0.64 (0.55, 0.76)	<0.0001	0.97 (0.72, 1.31)	0.85
Patient Region	Overall Test of Difference: *p* < 0.0001		Overall Test of Difference: *p* < 0.0001	
North Florida	1.00 (Ref)	N/A	1.00 (Ref)	N/A
South Florida	2.09 (1.78, 2.45)	<0.0001	0.82 (0.58, 1.17)	0.27
Central Florida	2.11 (1.77, 2.52)	<0.0001	0.46 (0.32, 0.65)	<0.0001
Patient Insurance Payer	Overall Test of Difference: *p* < 0.0001		Overall Test of Difference: *p* < 0.0001	
Commercial	1.00 (Ref)	N/A	1.00 (Ref)	N/A
Medicare	2.71 (2.13, 3.44)	<0.0001	1.48 (1.03, 2.12)	0.035
Medicaid	0.93 (0.72, 1.21)	0.58	0.36 (0.25, 0.52)	<0.0001
Other	0.66 (0.51, 0.87)	0.003	0.66 (0.39, 1.10)	0.11
Elixhauser Score	Overall Test of Difference: *p* < 0.0001		Overall Test of Difference: *p* = 0.0004	
No	1.00 (Ref)	N/A	1.00 (Ref)	N/A
Yes	0.60 (0.53, 0.68)	<0.0001	1.75 (1.38, 2.22)	<0.0001

Abbreviations: N/A, not applicable; OR, odds ratio; Ref, reference.
